# Multinucleate cell angiohistiocytoma

**DOI:** 10.1016/j.jdcr.2025.08.033

**Published:** 2025-09-18

**Authors:** Connor A. Sheehan, Jayci G. Rhein, Jordan T. Hyde, Jason B. Lee, Sylvia Hsu

**Affiliations:** a Department of Dermatology, Temple University Hospital, Philadelphia, Pennsylvania; b Department of Dermatology and Cutaneous Biology, Sidney Kimmel Medical College at Thomas Jefferson University, Philadelphia, Pennsylvania

**Keywords:** multinucleate cell angiohistiocytoma

## Case presentation

**Fig 1:**
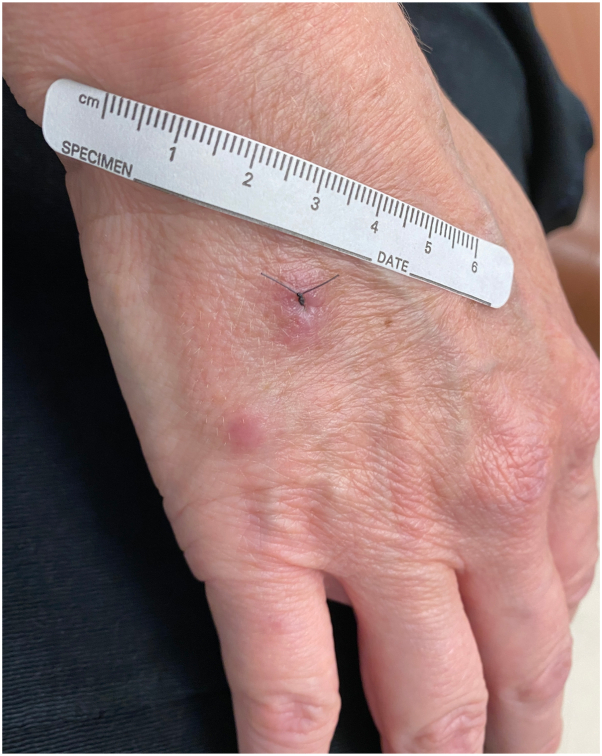
Two erythematous papules on the right dorsal hand.

**Fig 2:**
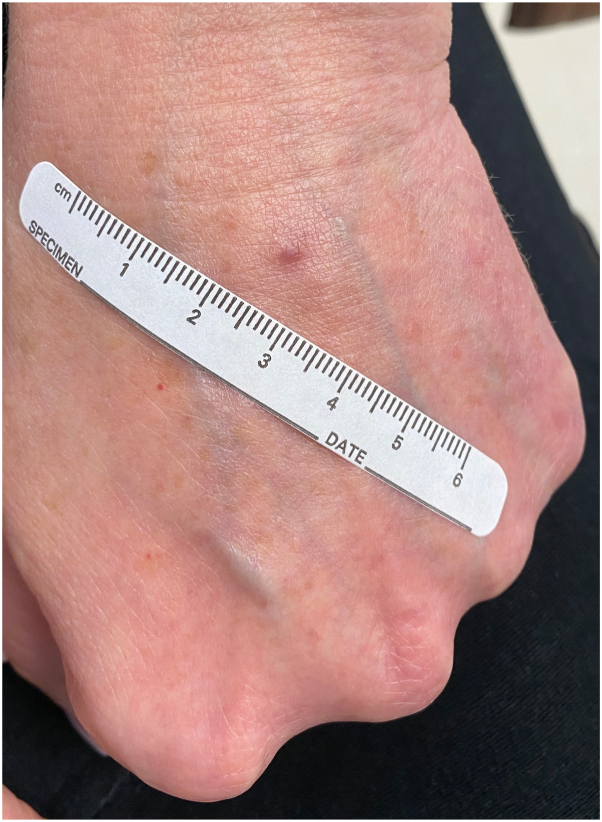
Erythematous papule on the left dorsal hand.

A 50-year-old woman presented with a 6-month history of papules on her hands. She had been evaluated by a previous dermatologist for these lesions and was given the diagnosis of scar. The patient had been vigilant about avoiding trauma to the hands yet noted the formation of new lesions. The papules were asymptomatic. On exam, there were 3 firm, nontender, nonscaly, erythematous papules on the bilateral dorsal hands (Figs 1 and 2).

**Question: Besides the dorsal hands, what is the most common location for this condition?**

A. Dorsal feet
B. Extensor surfaces
C. Face
D. Legs
E. Upper back and chest

## Answer discussion

**Fig 3:**
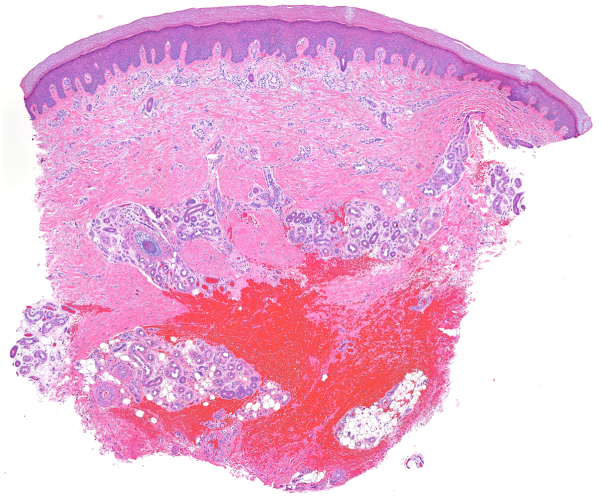
Dermal fibrosis and dilated vasculature with incidental procedural hemorrhage (H&E 5.6×).

**Fig 4:**
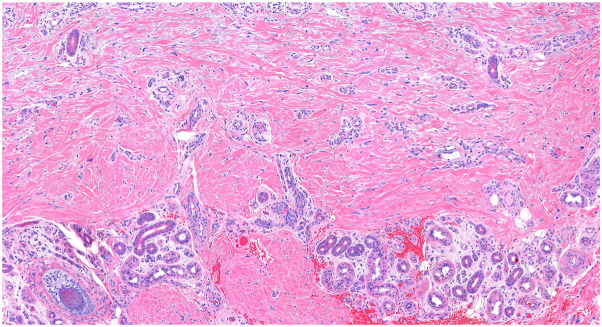
Thickened collagen bundles and dilated vessels with increased interstitial fibroblasts (H&E 15.6×).

**Fig 5:**
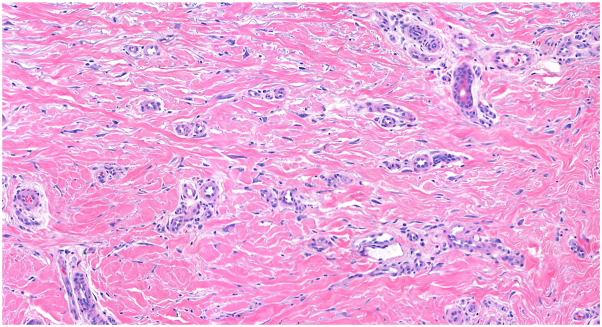
Characteristic multinucleated cells with scalloped borders (H&E 29.1×).

The correct answer is D. Legs. Multinucleate cell angiohistiocytoma (MCAH) is a benign condition characterized clinically by red or violaceous papules or plaques.^1^^,^^2^ It is a rare entity that has been described fewer than 200 times in the literature and is most commonly found on the dorsal hands or lower extremities of middle-aged women.^2^^,^^3^ The clinical appearance of MCAH can resemble that of angiofibroma, granuloma annulare, or Kaposi sarcoma, and biopsy is standard for making the diagnosis.^2^ Histologically, MCAH shows dermal fibrosis, histiocytosis, dilated vasculature, and stellate multinucleated cells (Fig 3, Fig 4, Fig 5). These giant cells are characteristic of this condition, although they are not pathognomonic.^1^ CD10 staining highlights the multinucleated cells but is not diagnostic.^1^ The pathogenesis is debated but MCAH is proposed to be a reactive fibroblast proliferation, possibly secondary to trauma, given distribution on trauma-prone sites.^1^^,^^4^^,^^5^ Some authors have proposed that this lesion may be a variant of dermatofibroma.^1^ There is some reported association of MCAH with estrogen receptor overexpression, which may explain the disproportionate incidence in female patients.^5^

The clinical behavior of MCAH is benign with some enlargement over time before either spontaneous regression or persistence. While there is no consensus treatment, case reports suggest efficacy of pulsed dye laser or surgical excision.^3^ Other reports include Nd:YAG, CO2 laser, intense pulsed light, and cryotherapy.^3^ Once treated, lesions are not expected to recur.^3^

## Conflicts of interest

None disclosed.
